# Exosomal LncRNA LINC00659 transferred from cancer-associated fibroblasts promotes colorectal cancer cell progression via miR-342-3p/ANXA2 axis

**DOI:** 10.1186/s12967-020-02648-7

**Published:** 2021-01-06

**Authors:** Lin Zhou, Jian Li, Yaping Tang, Mei Yang

**Affiliations:** 1 Departmemt of General Surgery, Xiangya Hospital, Central South University, Changsha, Hunan 410008 People’s Republic of China; 2 Department of Geriatric Medicine, Xiangya Hospital, Central South University, No. 87, Xiangya Road, Kaifu District, Changsha, Hunan 410008 People’s Republic of China

**Keywords:** Colorectal cancer, Cancer-associated fibroblasts, Exosomes, LINC00659, miR-342-3p, ANXA2, Proliferation, Invasion, Migration

## Abstract

**Background:**

Cancer-associated fibroblasts (CAFs) play a pivotal role in regulating tumor progression by transferring exosomes to adjacent cells. Our aim was to clarify the role of LINC00659 encapsulated in CAFs-derived exosomes (CAFs-exo) in colorectal cancer (CRC).

**Methods:**

CAFs and normal fibroblasts (NFs) were isolated and cultured. CAFs-exo and NFs-derived exosomes (NFs-exo) were characterized by transmission electron microscope and Western blot. The mRNA level of LINC00659 in CAFs-exo and NFs-exo were measured. Then we analyzed cell proliferation by CCK-8 and clone formation assay, cell migration by cell scratch, and cell invasion by Transwell. Epithelial mesenchymal transformation (EMT) related markers E-cadherin, N-cadherin, Vimentin and Snail-1 expressions were assessed by Western blot. The binding of LINC00659 and miR-342-3p, miR-342-3p and ANXA2 were analyzed by dual-luciferase reporter gene assay.

**Results:**

CAFs and NFs showed a spindle-like morphology. CAFs-exo promoted CRC cell proliferation, migration, invasion and EMT progression. The expression of LINC00659 in CAF-derived exosomes was significantly increased, and fibroblasts could transfer exosomal LINC00659 to CRC cells. We further revealed that transfection of miR-342-3p mimic or sh-ANXA2 could obviously reverse the promotion effect of exosomal LINC00659 on CRC progression. Functional studies reveal that LINC00659 is transferred from CAFs to the cancer cells via exosomes, where it promotes CRC cell proliferation, invasion, migration and EMT progression in vitro. Mechanistically, LINC00659 interacts directly with miR-342-3p to increase ANXA2 expression in CRC cells.

**Conclusion:**

Collected evidence supported that CAFs-derived exosomal LINC00659 promotes CRC cell proliferation, invasion and migration via miR-342-3p/ANXA2axis.

## Background

Colorectal cancer (CRC) is the third leading cancer worldwide and the fourth most prevalent neoplasm, with most rapidly increasing incidence and mortality [1]. At present, the five-year survival for CRC is only 64.9% [2]. Once CRC goes into advanced stage, surgical resection is useless and the prognosis of patients is extremely disappointing [3, 4]. Tumor progression is deeply influenced by the local microenvironment. The activated fibroblasts are often called cancer-associated fibroblasts (CAFs), which are currently identified as one of the most active cell types of the tumor microenvironment (TME) [5]. CAFs, as important components of the TME, can interact with cancer cells to facilitate tumorigenesis and progression [6]. Herein, with the unraveling of the relationship between CAFs and tumors, CAFs are considered as an important target for anti-cancer therapy.

Long noncoding RNAs (lncRNAs) represents a novel type of noncoding RNAs that greater than 200 nucleotides with limited protein-coding ability [7]. LINC00659, a long noncoding RNA, has been characterized as a novel oncogene that its expression level is considerably increased in CRC [8]. Although previous finding has supported the vital roles of LINC00659 in tumorigenicity, little is known about the upstream factors that induce the abundant expression of LINC00659 in CRC.

RNA molecules, such as lncRNAs, circular RNAs, miRNAs and mRNAs are excessively enriched in exosomes, which are secreted by multiple cell types [9, 10]. Accumulating evidences have revealed that exosomes are implicated in the dynamic crosstalk between CAFs and cancer cells that can shape the tumor environment, thus promoting tumor progression [11, 12]. Exosomes can serve as critical mediators between stroma intercellular and cancer cell communication via transferring genetic message associated contents in TME [6, 13]. Additionally, the ncRNA-loaded exosomes could mediate the TME of cancer cells by acting as mediators [14–16]. However, the mechanisms by which exosomes regulate LINC00659 expression and its functional role in CRC cells remain dismal. Fluorescence in Situ Hybridization (FISH) in this study found LINC00659 mainly located in the cytoplasm of CRC cells. Further experiments identified that lncRNA LINC00659 can be transferred from CAFs to CRC cells by exosomes. Therefore, we hypothesized that LINC00659 in CAFs-exo may exerts its function in CRC cells through ceRNA mechanism. This study was performed with the aim to explore the possible role and mechanism ofLINC00659 in CRC progression.

## Materials and methods

### Cell culture

The human CRC cell lines LOVO and SW48, obtained from American Type Culture Collection (ATCC, USA), were fostered in DMEM/F12 medium (Thermo Fisher Scientific, Wilmington, DE, USA) containing 10% fetal bovine serum (FBS; Thermo Fisher Scientific, Wilmington, DE, USA) in a 5% CO_2_ atmosphere at 37 °C.

### Isolation and culture of CAFs

Human CAFs (CRC group) and normal fibroblasts (NFs) (normal group) were acquired from fresh CRC and adjacent normal tissues. Tissue samples were collected from patients after written consents and approval of the Ethics Committee of Xiangya Hospital, Central South University were obtained. Part of the cells was used for detection of LINC00659 in CRC tissues using qRT-PCR and FISH. Tumor tissues and paired normal tissues were washed with sterile PBS. Then, the tissues were chopped into small pieces and digested with 0.1% of collagenase I (Sigma-Aldrich, Merck KGaA, Darmstadt, Germany) at 37 °C. The digested mixtures were centrifuged and rinsed in DMEM medium (Gibco, Grand Island, NY, USA) to remove the fat and tissue debris. Thereafter, tissues were fostered in DMEM medium (Gibco, Grand Island, NY, USA) containing 15% FBS for about 2 days. After the suspending cells and tissues were removed, the most adherent cells were fibroblasts, macrophages and epithelial cells. After 3 ~ 5 days of incubation, macrophages and epithelial cells were apoptotic with only fibroblasts saved. Fresh medium was replaced whenever deemed necessary. The primary fibroblasts isolated from tumor tissues were named “CAFs”, and from tumor paired normal tissues named “NFs”. Cells were used for study after 3 generations of culture and measured using microscopical measurement.

### Co-culture of fibroblasts and CRC cells

Transwell plates (Corning, NY, USA) were used to co-culture NFs and CAFs with CRC cells LoVo and SW48 [17]. NFs or CAFs were inoculated into the upper chamber while LoVo and SW48 were inoculated into the lower chamber at a density of 2 × 10^4^ cells/well. Exosomes secreted by fibroblasts could allow the migration of cells through the membrane into the lower chamber. After 24 h of co-culture, the collected CRC cells from the lower chamber were used for the following experiments. The experimental groups was divided into: Control group (no treatment), NFs group (Co-culture of NFs with LoVo/SW48) and CAFs group (Co-culture of CAFs with LoVo/SW48).

### Immunofluorescence

CAFs and NFs were seeded at 5000/well onto 24-well culture plates. Next, cells were fixed with 4% paraformaldehyde for 15 min, followed by 0.5% Triton-X-100 for 20 min. Thereafter, the cells were incubated with appropriate concentrations of primary antibody of α-SMA (19245S, 1:200, Cell Signaling Technology, Danvers, MA, USA), FAP (ab28244, 1:200, abcam, Cambridge, MA, USA), or Vimentin (5741S, 1:100, Cell Signaling Technology, Danvers, MA, USA) at 4 °C overnight, and then with Alexa Fluor 488-labeled second antibody goat anti-rabbit IgG (ab150077, 1:2000, abcam, Cambridge, MA, USA) away from light (45 min, room temperature). Next, the secondary antibody solution was removed and the cells were washed thrice by PBS (5 min) and then cultured with 0.5 µg/ml DAPI (5 min) at room temperature in the dark, followed by PBS wash (2 × 5 min). Finally, the immunofluorescence was visualized under a fluorescence microscope (BX61FL, OLYMPUS, Tokyo, Japan).

### Extraction and identification of exosomes

CAFs-derived exosomes (CAFs-exo) and NFs-derived exosomes (NFs-exo) were extracted by ultra-centrifugation: the supernatants from cell culture medium were collected and centrifuged at 4 °C at 2000 *g* for 30 min, 10,000 *g* for 40 min and 100,000 *g* for 70 min to separate deposited debris and supernatants. After washing twice with PBS, the deposited debris were centrifuged (100,000 *g*, 70 min, 4 °C) and subsequently resuspended in PBS and storage at − 80 °C.

Electron microscope observation: exosome suspensions were collected and then dripped onto a copper grid (1 min, room temperature). Thereafter, the exosomes were negatively stained with 3% (w/v) sodium phosphotungstate solution for 5 min, and washed under H_2_O and dried naturally at room temperature. Lastly, exosomes were observed and photographed using a transmission electron microscope (CM‐120, Philips, Eindhoven, Netherlands).

Exosome identification: the expressions of exosomal marker CD9, CD81 and TSG101 were monitored by flow cytometry (FCM) and Western blot.

FCM: 100 μl of exosome suspensions were incubated with appropriate primary antibody of CD9 (ab2215, 1:200, Abcam), CD81 (ab79559, 1:1000, Abcam), or TSG101 (ab83, 1:500, Abcam) for 30 min. Cells were further incubated for 30 min with FITC-conjugated secondary antibody goat anti-mouse IgG (ab6785, 1:1000, abcam). Exosomes were treated with RNase or RNase + TritonX-100 to detect LINC00659 expression.

### Extraction of exosomes

In brief, l μl of DiI (Santa Cruz Biotechnology, USA) was added to 100 μl exosome suspensions and the mixture was then incubated at 37℃ for 1 h in the dark. Exosomes were collected by ultra-centrifugation, and then resuspended in PBS before wrapped in foil and stored at -80℃. LoVo and SW48 cells were seeded into a 6-well plate (10^5^/well) and incubated at 37℃ in a 5% CO_2_ incubator overnight. The DiI-labeled exosomes were added into the 6-well plate after the cells were adhered to the wall. With further incubation for 24 h, LoVo and SW48 cells were stained with DAPI, and the exosomes were visualized and photographed via a fluorescence microscope (BX51, Olympus, Tokyo, Japan).

### Exosome inhibition experiments

To further verify the role of exosomes, exosome secretion was inhibited using Sphingomyelinase inhibitor GW4869 and 10 µM of GW4869 (Sigma-Aldrich, Merck KGaA, Darmstadt, Germany) was used to treat CAFs (designated GW4869-CAFs group), while Control group was treated with DMSO (designated DMSO-CAFs group). After 12 h, cells were washed thrice with sterile PBS before 10 ml of serum-free DMEM (Gibco, Grand Island, NY, USA) was added. CAFs were collected after 2 h.

### Cell transfection

pcDNA3.1-LINC00659, pcDNA3.1-ANXA2, pcDNA3.1, sh-LINC00659, miR-342-3p mimic, miR-342-3p inhibitor and their negative controls were obtained from Shanghai GenePharma Co. Ltd (Shanghai, China). All transfection was carried out using lipfectamine 2000 reagent (Invitrogen, Carlsbad, CA, USA) following the manufacturer’s protocol. The exosomes (from CAFs transfected with pcDNA3.1-LINC00659 or pcDNA3.1) incubated with CRC cells were named as pc-LINC00659-exo group or pc-exo group. The exosomes (from CAFs transfected with sh-LINC00659 or sh-NC) incubated with CRC cells were named as sh-LINC00659-exo group or sh-NC-exo group. sh-NC-exo was co-incubated with CRC and then transfected with miR-342-3p inhibitor or sh-ANXA2 plasmid were named as sh-NC-exo + in-miR-342-3p group or sh-NC-exo + sh-ANXA2 group. sh-LINC00659-exo was co-incubated with CRC and then transfected with miR-342-3p inhibitor or pcDNA3.1-ANXA2 plasmid were named as sh-LINC00659-exo + in-miR-342-3p group or sh-LINC00659-exo + pc-ANXA2 group.

### qRT-PCR

Total RNAs were extracted utilizing TRIzol reagent (Invitrogen, Carlsbad, CA, USA). Reverse transcription was carried out through a reverse transcription kit (TaKaRa, Tokyo, Japan) based on the manufacturer’s guidance. Gene expression was detected with the LightCycler 480 (Roche, Indianapolis, IN, USA) fluorescence quantitative PCR instrument. The reaction was performed under the guidance of the fluorescent quantitative RT-PCR kit (SYBR Green Mix, Roche Diagnostics, Indianapolis, IN). The thermocycling program were as follows: 10 s denaturation at 95˚C, followed by 45 cycles of 5 s denaturation at 95˚C, 10 s annealing at 60˚C and 10 s extension at 72˚C, and a final extension step of 5 min at 72 °C. The qPCR was repeated thrice. GAPDH and U6 were used as the internal reference of mRNA and miRNA expressions, respectively. Data were calculated by 2^−ΔΔCt^ method, with the following formula: ΔΔCt = [Ct _(target gene)—_Ct _(reference gene)_] _experimental group—_[Ct _(target gene)—_Ct _(reference gene)_] _control group_. The primer sequences for all candidate reference genes are depicted in Table 1.

**Table 1:** Primer sequence for quantitative reverse transcription polymerase chain reaction to determine the expression levels of E-cadherin, N-cadherin, Vimentin, and Snail-1, U6, LINC00659, ANXA2, GAPDH

Name of primer	Sequences
E-cadherin-F	CGTCGAGCTCTTGACCGAAA
E-cadherin-R	TCAAACACCTCCTGTCCTCT
N-cadherin-F	AGGGGAGAGGTGCTCTACTG
N-cadherin-R	GGGGTAATCCACACCACCTG
Vimentin-F	TCCGCACATTCGAGCAAAGA
Vimentin-R	TGAGGGCTCCTAGCGGTTTA
Snail-1-F	CGAGCCATAGAACTAAAGCC
Snail-1-R	TGAGGGAGGTAGGGAAGTG
U6-F	CTCTCGCTTCGGCAGCACA
U6-R	ACGCTTCACGAATTTGCGT
LINC00659-F	ACCCCTGAAGGACCATATCCA
LINC00659-R	GGCTCGGCTGTGTCTCAAG
ANXA2-F	CTCTACACCCCCAAGTGCAT
ANXA2-R	TCAGTGCTGATGCAAGTTCC
GAPDH-F	GCAAGGATGCTGGCGTAATG
GAPDH-R	TACGCGTAGGGGTTTGACAC

*F* forward, *R* reverse

### Western blot analysis

Cells were lysed by RIPA buffer (Beyotime Institute of Biotechnology, China) and protein samples were acquired. After protein concentration were determined by BCA kit (Beyotime Institute of Biotechnology, China), the corresponding volume of protein was mixed into the loading buffer, followed by heated in boiling water bath for 3 min to minimize denaturation. The electrophoresis was initially run at 80 V for 30 min, then 120 V for 1– 2 h. After being transferred on an ice bath at 300 mA for 60 min, the membrane was rinsed for 1– 2 min, and then sealed in blocking buffer for 60 min at room temperature or cultured overnight at 4˚C. Primary antibody of FAP (ab28244, 1:1000), CD9 (ab2215, 1:500), CD81 (ab79559, 1:1000), TSG101 (ab83, 1:1000), ANXA2 (ab41803, 1:1000) (all from abcam, Cambridge, MA, USA), α-SMA (19245S, 1:1000), E-cadherin (3195S, 1:1000), N-cadherin (13116S, 1:1000), or Vimentin (5741S, 1:1000) (Cell Signaling, Boston, USA) was incubated for 1 h at room temperature. The membrane was washed with PBS thrice (10 min each), and then incubated with secondary antibodies of HRP-marked goat anti-rabbit IgG (1:5000, Beijing ComWin Biotech Co., Ltd., Beijing, China) for 1 h at room temperature and washed repeatedly (3 × 10 min). Finally, the signals were detected by chemiluminescence imaging system (Bio-rad).

### Cell counting kit-8 (CCK-8) assay

The transfected cells were seeded at density of 2 × 10^3^ cells/well in 96-well plates. Triplicates were conducted and cells were fostered in a 5% CO_2_ incubator at 37˚C. Cell viability was measured utilizing Cell Counting Kit (CCK-8, Tokyo, Dojindo, Japan). Afterwards, 10 μL of CCK-8 solution was added into each well, followed by incubated for 2 h at 37 °C in 5% CO_2_. The culture medium was then removed and the plates were washed twice by PBS. Finally, the plates were detected at 450 nm wavelength to obtain absorbance value (OD value). The average OD value for each sample tested was performed thrice.

### Colony formation assay

After transfection for 24 h, the collected cells of each group were trypsinized and centrifuged for 5 min at 1500 rpm at 25 °C and then resuspended in complete medium. Thereafter, cells were seeded into 6-well plates (500 cells/well) containing 2 ml of complete medium and were maintained at 37 °C in an atmosphere of 5% CO_2_ for 2 to 3 weeks. When clones were visible to the naked eye, the culture was terminated and the culture medium was removed. Cells were rinsed in PBS for two times and then fixed with 1.5 ml of formaldehyde for 15 min. Afterwards, cells were stained with 1 ml of Giemsa solution in the dark for 20 min. After that, Giemsa solution was slowly washed away with running water. The plates were air-dried in an inverted position and the number of cells was counted.

### Cell scratch test

Cell scratch test was conducted as described previously [18]. In brief, the cells in the control and the experiment groups were plated in six-well plates. When cells were achieved 90% confluence, three scratches were drawn in the plate with a 100 μL pipette tip. Afterwards, cells were washed with PBS, followed by replaced with serum-free medium and incubated for 24 h. The gap between cells was photographed under a low-magnification phase-contrast microscope (Olympus MK, Tokyo, Japan).

### Transwell assay

Matrigel test: A chamber coated with Matrigel stored at -20℃ was removed and melted at room temperature. Next, serum-free medium (0.5 ml) was added into Transwell chamber (coring, New York, USA) and then plated in 24-well plate for 2 h in 5% CO_2_ at 37 °C until the nutrient was fully absorbed. Thereafter, Cells (1 × 10^5^) were inoculated into Transwell chamber in which the culture medium containing 1% FBS was added to 500 µL, and 0.75 ml of culture medium containing 15% FBS was then added into the lower chamber, followed by incubated at constant temperature and humidity (37℃, 5% CO_2_) for 20 h. Then, the culture medium on the upper chamber was discarded. After being washed two times with PBS (calcium-free), the cells were then fixed 10 min with 4% paraformaldehyde. After being washed with PBS twice (2 min each), the cells were stained with crystal violet for 10 min. Cells and the upper Matrigel layer were removed and rinsed with PBS thrice. Cells were visualized and counted from six randomly selected fields under a microscope (200 × magnification).

### Expression data from the cancer genome atlas

The Transcriptome expression profile of CRC was downloaded from TCGA database (http://gepia.cancer-pku.cn/index.html). A total of 275 cases of CRC tissues and 348 cases of adjacent normal tissues were obtained. The criteria for selection were set at |Log2FC|> 1, *p* value < 0.01, among which the data for 370 cases of CRC patients were included for survival analysis.

### Dual-luciferase reporter gene assay

The binding sites of LINC00659 and miR-342-3p, miR-342-3p and ANXA2 were performed by the online software Starbase 2.0 (http://starbase.sysu.edu.cn/index.php). The mutated and wild-type biding sites for LINC00659 and ANXA2 were designed based on the prediction results. Then, the binding sequences were cloned into luciferase reporter gene vector (pGL3-Basic), and the recombined vector was co-transfected with 30 nM of miR-342-3p mimic, miR-342-3p inhibitor, or their negative controls into HEK-293 T cells, respectively. After that, the activities of firefly luciferase and Renilla luciferase in each well were assessed. Activity of renilla luciferase was used as an internal reference, and the relative activity of luciferase was the ratio of firefly luciferase to Renilla luciferase activities.

### Statistical analysis

GraphPad Prism 6.0 (GraphPad Software Inc.) and SPSS 18.0 (IBM Corp., Armonk, NY, USA) were used for data analysis. Continuous data were expressed as mean ± standard deviation. Two groups were compared utilizing *t* test, and one-way analysis of variance (ANOVA) was applied to determine the comparison among multiple groups. *P* < 0.05 was deemed as statistically significant.

## Results

### Characteristics of CAFs-exo and NFs-exo from patients with CRC

**Fig. 1:**
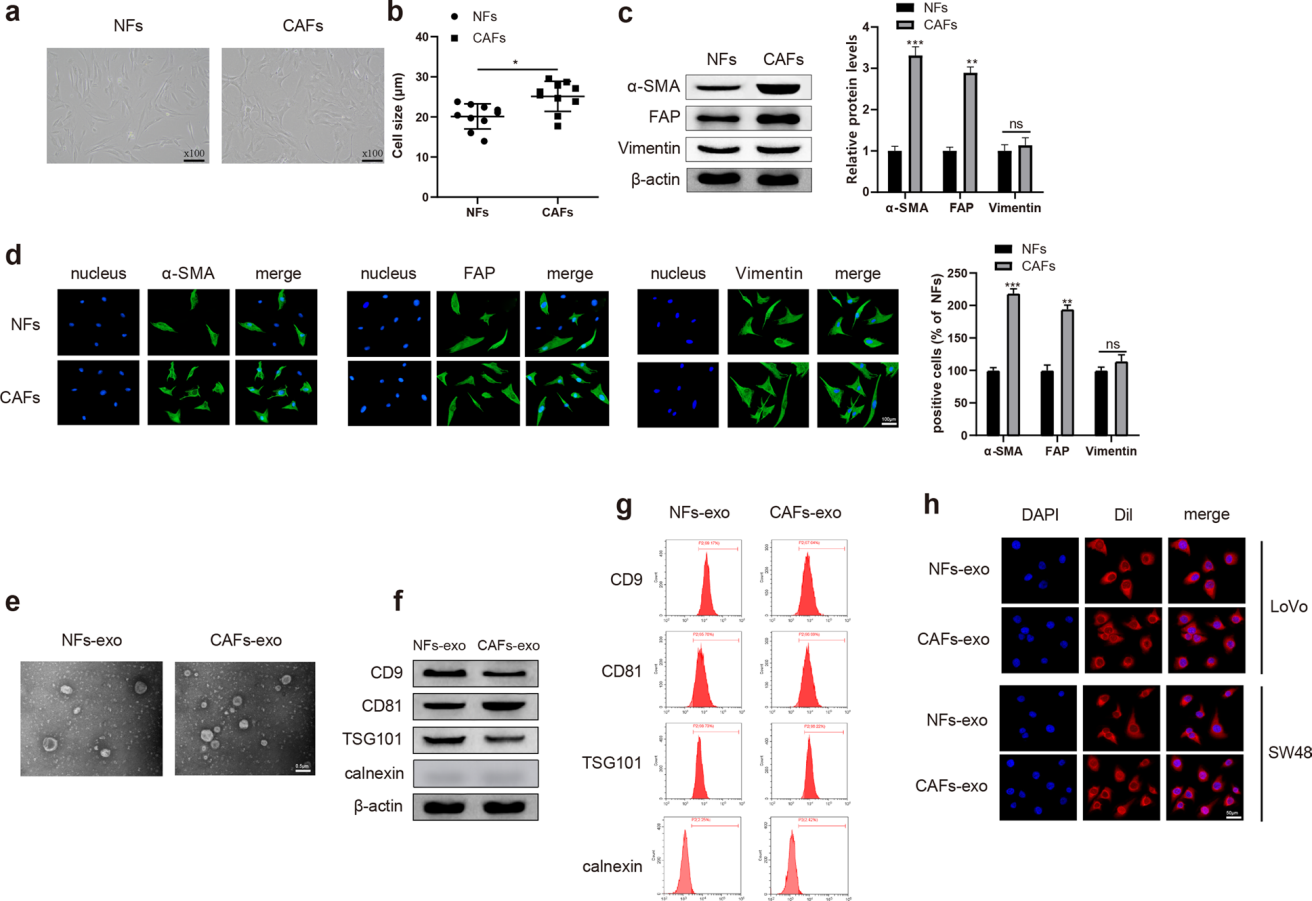
Characteristics of CAFs-exo and NFs-exo from patients with CRC. Phenotypes and morphologies of CAFs and NFs were observed using a laser confolcal microscope (**a**). Microscope graticules for measurement of CAFs and NFs (**b**); Western blot and immunofluorescence were used to detect the levels of α-SMA, FAP and Vimentin, bars = 100 μm (**c**, **d**). TEM was applied to detect CAFs-exo and NFs-exo, bars = 20 μm (**e**). The levels of CD9, CD81 and TSG101 were monitored by Western blot and and FCM (**f**–**g**). DiI-labeled exosomes were visualized via a fluorescence microscope (**h**). N = 3, **P < 0.01, ***P < 0.001 vs NFs group; *CAFs-exo* CAFs-derived exosomes, *NFs-exo* NFs-derived exosomes, *CRC* colorectal cancer, *TEM* transmission electron microscopy, *FCM* flow cytometry

Primary CAFs and NFs were isolated from patients with CRC. Then, phenotypes and morphologies of CAFs and NFs were observed. Under a laser confocal microscope, we found CAFs and NFs showed a spindle-like morphology (Fig. 1a). The microscopical measurement demonstrated CAFs were larger than NFs (Fig. 1b , *P*< 0.05). Previous literature has reported that the viability of CAFs is higher compared with that of NFs [19], but no obvious difference in the expression of activation markers α-SMA between CAFs and NFs has been revealed [20]. Herein, we performed Western blot to further analyze the activation of CAFs and NFs (Fig. 1c). The results presented that the expressions of well-recognized markers α-SMA and FAP were higher in CAFs than in NFs; however, the expression of Vimentin was not different between the two groups. These biomarker expression patterns were confirmed using immunofluorescence (Fig. 1d). We disclosed that the positive rates of α-SMA and FAP were much higher in CAFs than in NFs, whilst there was no obvious difference in the positive rate of Vimentin between the two groups. Collective results concluded that the purified CAFs and NFs is qualified and can be used for the subsequent analysis.

We then isolated the exosomes secreted by CAFs and NFs. The exosomes were observed by electron microscope, as shown in Fig. 1e. Western blot and FCM were applied to measure the expressions of the exosomal marker CD9, CD81, TSG101 and Calnexin. The results manifested that CD9, CD81 and TSG101 were positively expressed in CAFs-exo and NFs-exo (**Fig. ****1****f, g** , *P*< 0.05), but Calnexin was negative in CAFs-exo and NFs-exo. After LoVo and SW48 cells were incubated with DiI-labeled CAFs-exo or NFs-exo, red fluorescence was localized in the cytoplasm of CRC cells (Fig. 1h), while no red fluorescence can be observed in PBS treated LoVo and SW48 cells (Additional file 1: Figure S1), indicating exosomes were ingested by LoVo and SW48 cells. The above results indicated that the extracted exosomes meet the requirement for the following experiments.

### CAFs promote CRC cell progression by transferring exosomes

**Fig. 2:**
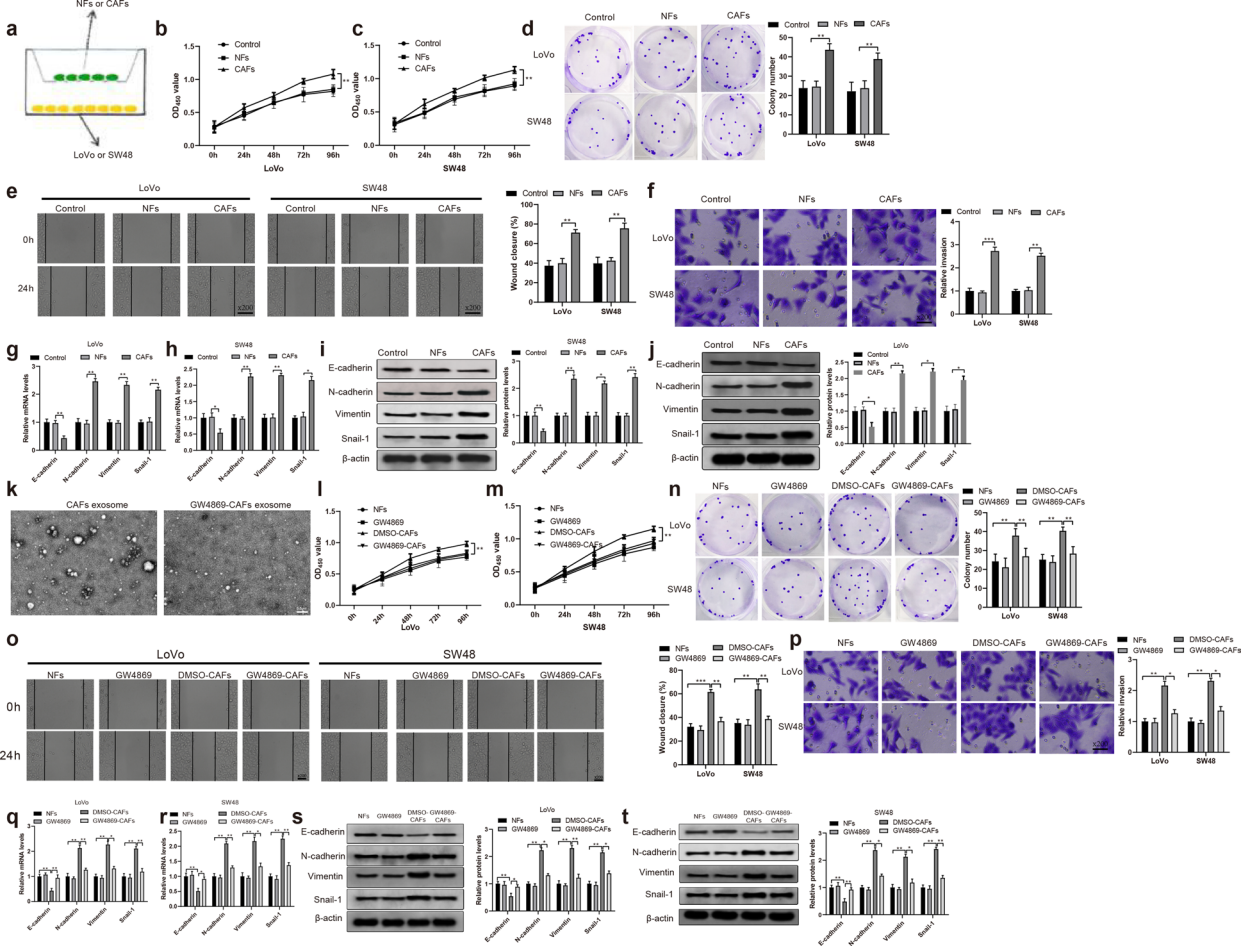
CAFs and NFs on CRC cell progression. CAFs and NFs were separately co-cultured with LoVo and SW48 cells (**a**). Cell proliferation was analyzed by CCK-8 (**b**, **c**) and clone formation assay (**d**), cell migration by cell scratch (**e**), and cell invasion by Transwell (**f**). qRT-PCR (G-H) and Western blot (i. j) were performed to test the levels of EMT related markers. *P < 0.05, **P < 0.01, vs NFs group. Transmission electron microscope was applied to observe the secretion of exosomes in CAFs (**k**). After cells were treated with exosomal inhibitor GW4869, cell proliferation, cell migration, cell invasion and EMT related markers were measured by CCK-8 (**l**–**m**) and clone formation assay (**n**), cell scratch (**o**), Transwell (**p**), qRT-PCR (**q**, **r**) and Western blot (**s**, **t**), respectively. N = 3, *P < 0.05, **P < 0.01, ***P < 0.001 vs DMSO-CAFs group; *CAFs* cancer-associated fibroblasts, *NFs* normal fibroblasts, *CRC* colorectal cancer, *EMT* epithelial mesenchymal transformation

We then tested the functional role of CAFs in LoVo and SW48 cells. As depicted in Fig. 2a, CAFs and NFs were separately co-cultured with LoVo and SW48 cells for 24 h. Then, CCK-8 and clone formation assay illustrated elevated proliferation and invasion ability in CAFs group (vs. NFs group) (Fig. 2b–d , *P*< 0.05). Additionally, higher migration rate and invasion ability were also found in CAFs group than in NFs group, detected by cell scratch (Fig. 2e , *P*< 0.05) and Transwell (Fig. 2 f, *P*< 0.05), respectively. Furthermore, the detection of qRT-PCR and Western blot revealed that the levels of epithelial mesenchymal transformation (EMT) related markers N-cadherin, Vimentin and Snail-1 were significantly heightened while E-cadherin was obviously inhibited in CAFs group (vs. NFs group) (Fig. 2g–j , *P*< 0.05). There was no difference in proliferation, migration, invasion and EMT related markers between the Control group and the NFs group. Collective data presented that CAFs could enhance LoVo and SW48 cell progression.

To further verify the function of CAFs in CRC, CAFs was treated with exosomal inhibitor GW4869, and then co-cultured with LoVo and SW48 cells. Transmission electron microscope demonstrated that GW4869 inhibits the exosomes secreted by CAFs (Fig. 2k). The detection presented that GW4869-CAFs group had distinctly lower abilities of cell proliferation (Fig. 2l–n , *P*< 0.05), migration (Fig. 2o , *P*< 0.05) and invasion (Fig. 2p , *P*< 0.05), and meanwhile increased levels of E-cadherin in addition to decreased levels of N-cadherin, Vimentin and Snail-1 (Fig. 2q–t  *P*< 0.05), compared with DMSO-CAFs group. Comparisons between NFs and GW4869 groups showed no significant difference. The above data disclosed that CAFs could promote CRC cell proliferation, migration, invasion and EMT progression by secreting exosomes.

### CAFs-derived exosomal LINC00659 promotes CRC cell progression

**Fig. 3:**
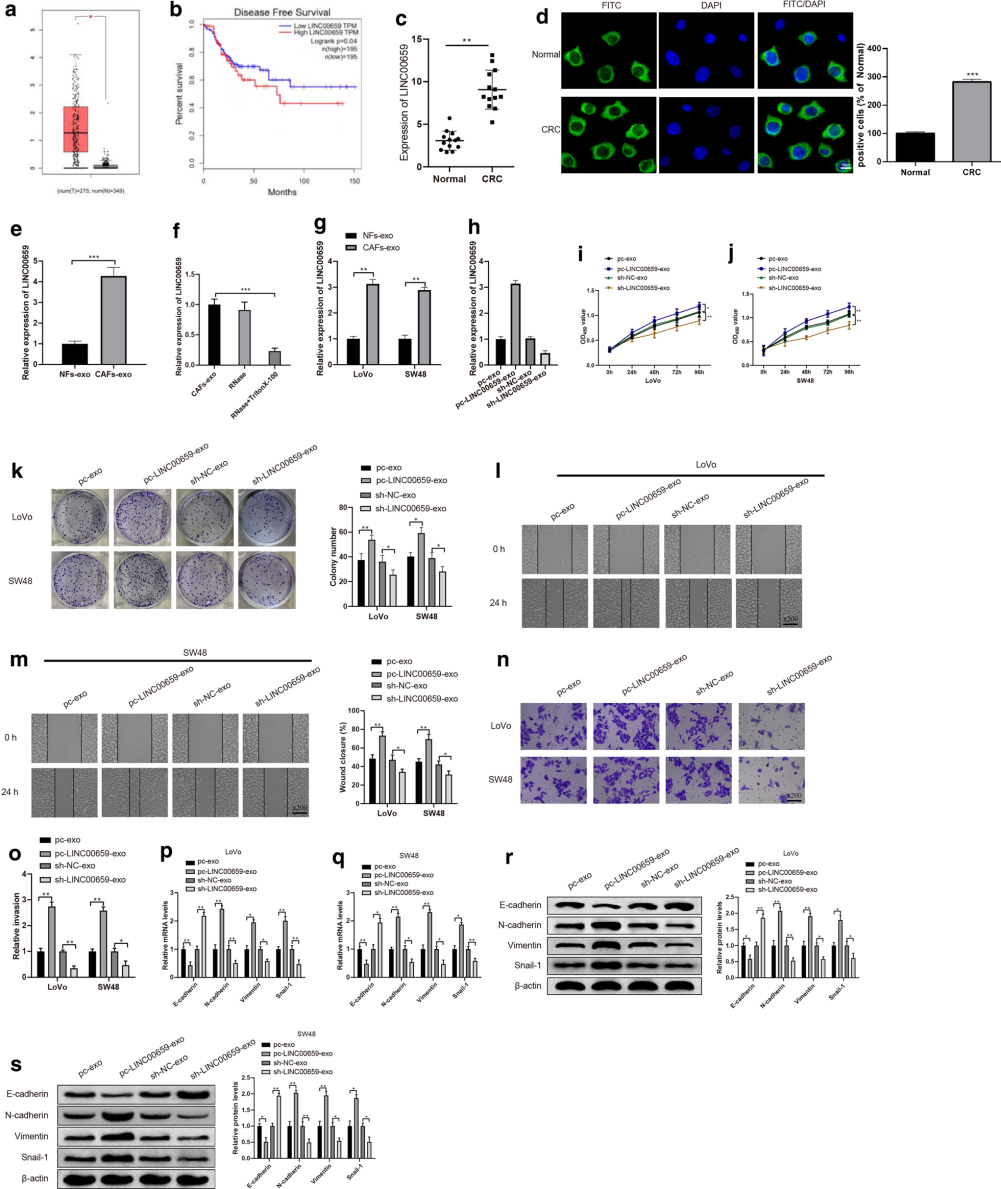
CAFs-derived exosomal LINC00659 promotes CRC cell progression. Note: TCGA database shows expression of LINC00659 is associated with prognosis and progression in CRC (**a**, **b**). qRT-PCR and FISH were conducted to measure the expressions of LINC00659 in CRC and adjacent cancer tissues (**c**, **d**). The mRNA expression of exosomal LINC00659 transferred from NFs and CAFs were monitored by qRT-PCR (**e**). After CAFs-exo was treated with RNase and TritonX-100, qRT-PCR was used to assess the level of CAFs-derived exosomal LINC00659 (**f**). Following CAFs-exo and NFs-exo were co-cultured with CRC cells, the level of LINC00659 was measured by qRT-PCR (**g**). Exosomes were extracted after transfection of overexpressed or silenced LINC00659 plasmid in CAFs, the expression of exosomal LINC00659 was analyzed by qRT-PCR (**h**). Cell proliferation was analyzed by CCK-8 (**i**–**j**) and clone formation assay (**k**), cell migration by cell scratch (**l**–**m**), and cell invasion by Transwell (**n**, **o**). qRT-PCR (**p**, **q**) and Western blot (**r**–**s**) were performed to test the levels of EMT related markers. N = 3, *P < 0.05, **P < 0.01, ***P < 0.001; *CAFs-exo* CAFs-derived exosomes, *NFs-exo* NFs-derived exosomes, *CRC* colorectal cancer, *EMT* epithelial mesenchymal transformation

LINC00659 expression in CRC tissues was analyzed in TCGA database. The results displayed that LINC00659 levels in CRC tissues were stronger than in adjacent cancer tissues (Fig. 3a , *P*< 0.05) and further analysis found that high expression of LINC00659 predicted a relatively poor prognosis while low expression of LINC00659 is associated with better prognosis (Fig. 3b , *P*< 0.05)*.* Besides, qRT-PCR and FISH results on both clinical CRC tissues and adjacent normal tissues presented that LINC00659 was expressed more frequently in CRC group than in Normal group (Fig. 3c, d , *P*< 0.05). Results of qRT-PCR manifested that LINC00659 was strongly expressed in CAFs-exo than in NFs-exo (Fig. 3e , *P*< 0.05). LINC00659 expression had no obvious change in CAFs-exo following RNase treatment, while treatment with RNase and TritonX-100 lead to inhibited LINC00659 expression (Fig. 3f , *P*< 0.05), which hinted that LINC00659 is wrapped in membrane. Further experiments found that co-culture of CAFs-exo with LoVo and SW48 cells had distinctly elevated LINC00659 expression, compared to NFs-exo (Fig. 3g , *P*< 0.05)*.* In a word, the above findings showed that LINC00659 was enriched in CAFs-exo to promote CRC cell progression.

To further confirm CAFs-exo promote CRC progression including proliferation, migration and invasion by transferring LINC00659, we separately transfected pcDNA3.1-LINC00659 and sh-LINC00659 in CAFs and then extracted the exosomes in CAFs. The detection manifested that the expression of LINC00659 in the pcDNA3.1-LINC00659 group was enhanced, while it was weakened in the sh-LINC00659 group compared with their negative controls (Fig. 3h , *P*< 0.05)*.* Then, after transfected with pcDNA3.1-LINC00659 or sh-LINC00659, LoVo and SW48 cells were incubated with CAFs-exo. The detection revealed that sh-LINC00659-exo group had decreased abilities of cell proliferation (Fig. 3i–k  , *P*< 0.05)*,* migration (Fig. 3l, m , *P*< 0.05) and invasion (Fig. 3n, o , *P*< 0.05) when compared with that in the sh-NC-exo group in LoVo and SW48 cells, whereas pc-LINC00659-exo group behaved in the opposite fashion (vs. pc-exo group) (Fig. 3i–o , *P*< 0.05)*.* Moreover, the detection of EMT related markers presented that the expressions of N-cadherin, Vimentin and Snail-1 were effectively repressed whilst E-cadherin was strongly expressed in the sh-LINC00659-exo group (vs. sh-NC-exo group), however, reversed expression patterns were found in pc-LINC00659-exo when compared with pc-exo group (Fig. 3p–s , *P*< 0.05). These data suggested that LINC00659 was enriched in CAFs-exo and promoted the development of CRC cells.

### CAFs-derived exosomal LINC00659 acts as a sponge for miR-342-3p to regulate ANXA2 expression

**Fig. 4:**
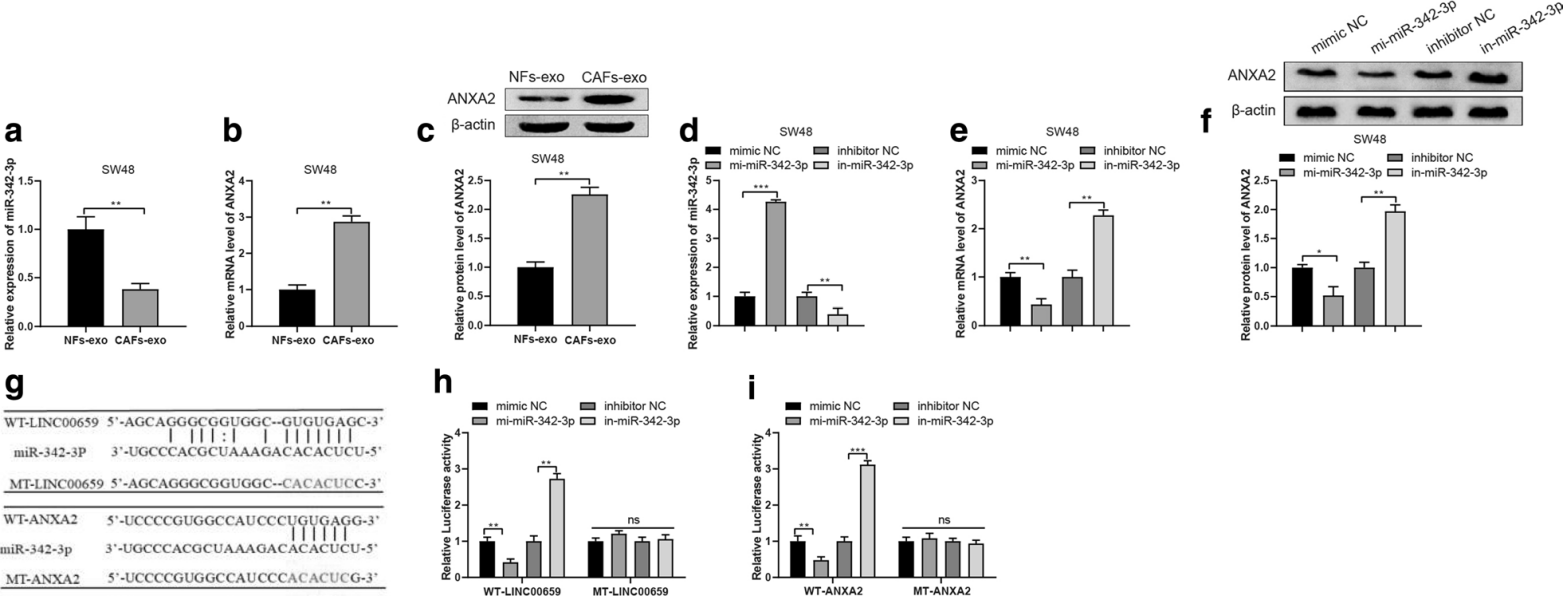
CAFs-derived exosomal LINC00659 acts as a sponge for miR-342-3p to regulate ANXA2 expression. Note: After NFs-exo and CAFs-exo were co-incubated with SW48 cells, qRT-PCR was applied to measure miR-342-3p expression in SW48 cells (**a**). The levels of ANXA2 in SW48 cells were assessed by qRT-PCR and Western blot, respectively (**b**, **c**). Following transfection of miR-342-3p mimics or inhibitors in SW48 cells, the expression of miR-342-3p in SW48 cells were measured by qRT-PCR (**d**). The levels of ANXA2 in SW48 cells were detected by qRT-PCR and Western blot, respectively (**e**, **f**). Prediction of binding sites and designed mutation sites of LINC00659 and ANXA2, LINC00659 and miR-342-3p was performed by the online software Starbase (**g**). Dual luciferase reporter assay was used to test the interactions of LINC00659 and miR-342-3p (**h**) as well as ANXA2 and miR-342-3p (i). N = 3, *P < 0.05, **P < 0.01, ***P < 0.001; *CAFs-exo* CAFs-derived exosomes, *NFs-exo* NFs-derived exosomes

In the next step, we sought to explore the mechanism of CAFs-derived exosomal LINC00659 in CRC. Then, NFs-exo or CAFs-exo was co-cultured with SW48 cells, the detection manifested that miR-342-3p was lowly expressed (Fig. 4a, *P* < 0.01) while the levels of ANXA2 were upregulated significantly (Fig. 4b, c , *P*< 0.05), suggesting that CAFs-exo could inhibit miR-342-3p and enhance ANXA2 expression.

Next, miR-342-3p mimic, miR-342-3p inhibitor or their negative controls was transfected into SW48 cells. Compared with mimic NC group, mi-miR-342-3p group increased the levels of miR-342-3p (Fig. 4d , *P*< 0.01) but decreased the levels of ANXA2 (Fig. 4e, f , *P*< 0.05). However, reversed expression patterns were observed in the in-miR-342-3p group when compared with inhibitor NC group. These data indicated that miR-342-3p negatively regulates ANXA2.

Searching from online software Starbase, the designed mutation sites and predicated binding sites of miR-342-3p and LINC00659, miR-342-3p and ANXA2 are shown as Fig. 4g. Then, luciferase reporter gene assay disclosed that miR-342-3p potentially binds with LINC00659 (Fig. 4h , *P*< 0.05) and ANXA2 (Fig. 4i  *P*< 0.05). The above data displayed that CAFs-derived exosomal LINC00659 competes with ANXA2 for binding with miR-342-3p.

### CAFs-derived exosomal LINC00659 promotes CRC cell progression via downregulating miR-342-3p

**Fig. 5:**
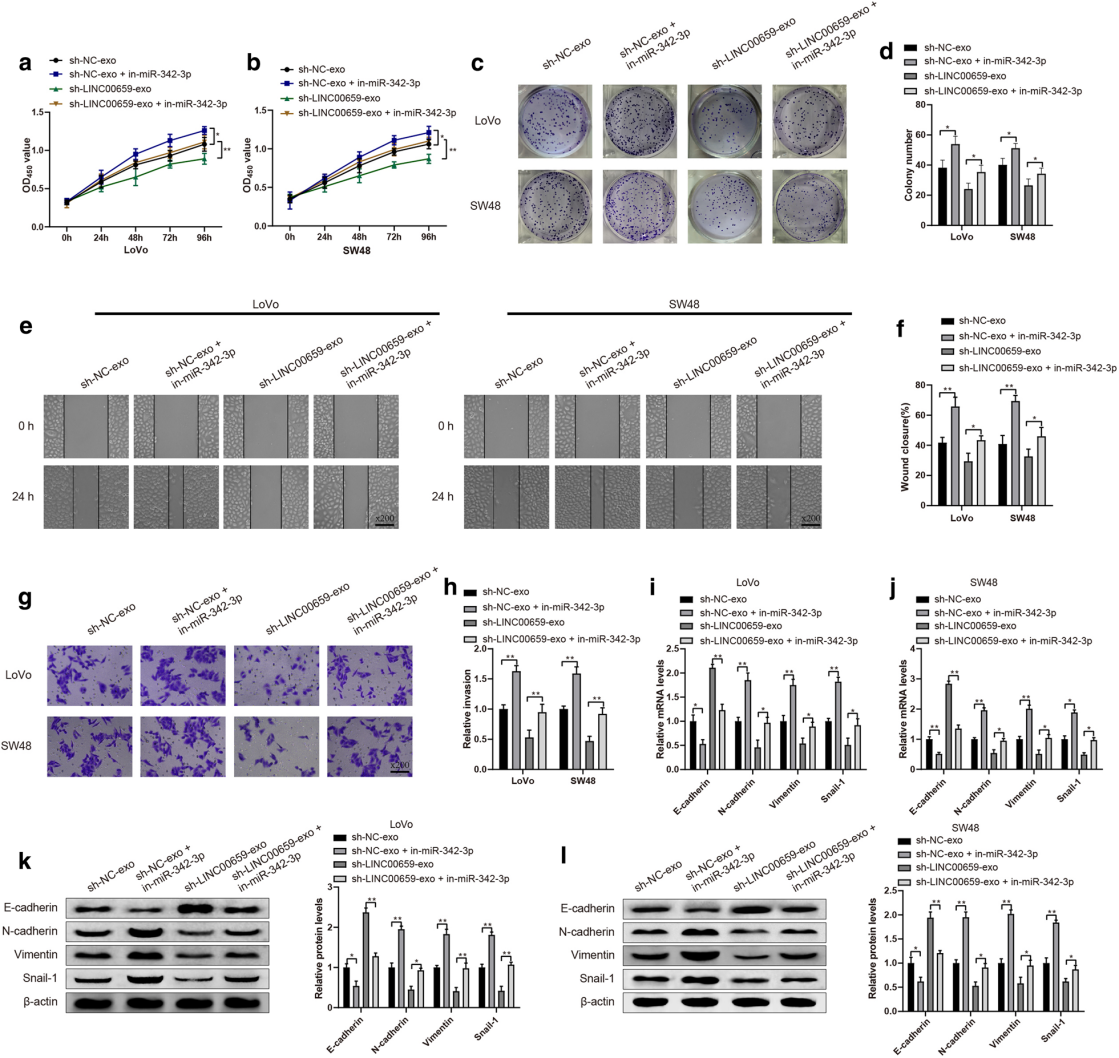
CAFs-derived exosomal LINC00659 promotes CRC cell progression via blocking miR-342-3p. Note: Cell proliferation, cell migration, cell invasion and EMT related markers were analyzed by CCK-8 (**a**, **b**) and clone formation assay (**c**, **d**), cell scratch (**e**, **f**), Transwell (**g**, **h**), qRT-PCR (**i**, **j**) and Western blot (**k**, **l**), respectively. N = 3, *P < 0.05, **P < 0.01; *CAFs* cancer-associated fibroblasts, *CRC* colorectal cancer, *EMT* epithelial mesenchymal transformation

This step was used to investigate whether CAFs-derived exosomal LINC00659 promoted CRC cell progression by downregulating miR-342-3p. The expression patterns of EMT related markers including E-cadherin, N-cadherin and Vimentin, in addition to cell development were assessed. The detection presented that cell proliferation (Fig. 5a–d , *P*< 0.05), migration (Fig. 5e, f , *P*< 0.05) and invasion (Fig. 5g, h , *P*< 0.05) were elevated, however, the levels of E-cadherin were distinctly inhibited whilst the levels of N-cadherin, Vimentin and Snail-1 (Fig. 5i–l , *P*< 0.05) were remarkably enhanced in sh-NC-exo + in-miR-342-3p group, compared with sh-NC-exo group. Similar expression patterns were found in sh-LINC00659-exo + in-miR-342-3p group (vs. sh-LINC00659-exo group). Combining with the aforementioned researches, CAFs-derived exosomal LINC00659 was confirmed to promote CRC cell progression by sponging miR-342-3p.

### CAFs-derived exosomal LINC00659 promotes CRC cell progression via upregulating ANXA2

**Fig. 6:**
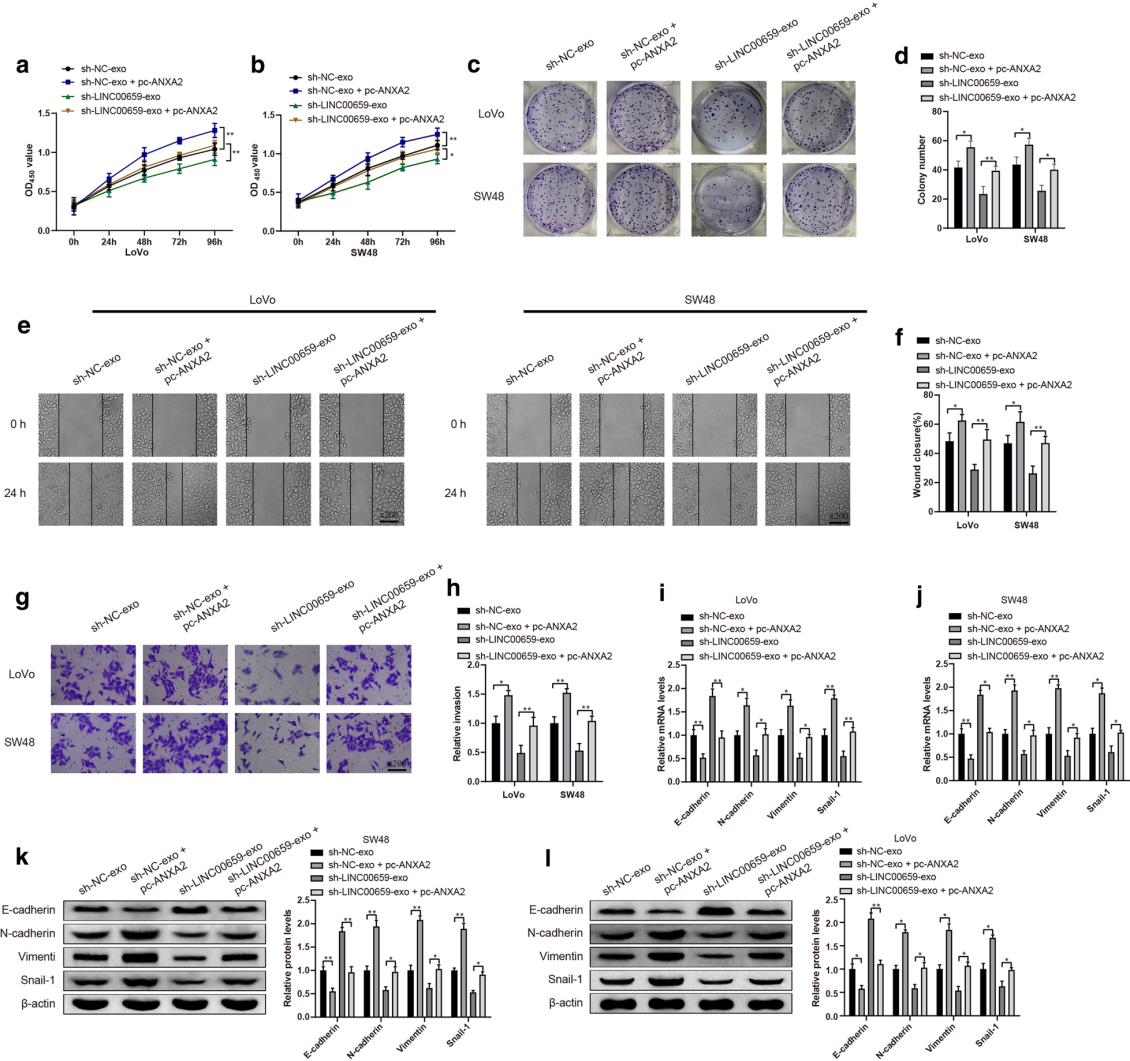
CAFs-derived exosomal LINC00659 promotes CRC cell progression via upregulating ANXA2. Note: Cell proliferation, cell migration, cell invasion and EMT related markers were analyzed by CCK-8 **a**, **b** and clone formation assay **c**, **d**, cell scratch (**e**, **f**), Transwell (**g**, **h**), qRT-PCR (**i**, **j**) and Western blot (**k**, **l**), respectively. N = 3, *P < 0.05, **P < 0.01; *CAFs* cancer-associated fibroblasts, *CRC* colorectal cancer, *EMT* epithelial mesenchymal transformation

We further investigated whether CAFs-derived exosomal LINC00659 facilitated CRC cell development via upregulation of ANXA2. The levels of EMT related markers, in addition to cell development were tested by qRT-PCR and Western blot assay. According to the results, sh-NC-exo + pc-ANXA2 group had distinctly elevated abilities of cell proliferation (Fig. 6a–d , *P*< 0.05), migration (Fig. 6e, f , *P*< 0.05) and invasion (Fig. 6g, h , *P*< 0.05), and meanwhile reduced levels of E-cadherin coupled with increased levels of N-cadherin, Vimentin and Snail-1 (Fig. 6i–l , *P*< 0.05) in LoVo and SW48 cells, compared with sh-NC-exo group. Similar expression patterns were found in sh-LINC00659-exo + pc-ANXA2 group (vs. sh-LINC00659-exo group). The aforementioned data suggested that CAFs-derived exosomal LINC00659 upregulates ANXA2 to promote CRC cell progression.

## Discussion

Firstly we identified LINC00659 was highly expressed in CAF-exo than that in NF-exo. Meanwhile, evidence in a previous study and TCGA database showed increased expression of LINC00659 in CRC tissues. Therefore those observations intrigued us to further explore the mechanism of LINC00659 in CRC. In the present work, we uncovered that, for the first time, exosomes from CAFs can transfer LINC00659 to CRC cells and are correspondingly associated with CRC cell development. Further, CRC tumor expresses obviously higher levels of lncRNA LINC00659 than do normal cells, and the mechanism herein illustrates that exosomal LINC00659 functioning as a ceRNA mediates miR-342-3p/ANXA2 axis, thereby promoting cell proliferation, invasion, migration and EMT in CRC.

In our study, GW4869 was used to inhibit the secretion of exosomes in CAFs. GW4869 is a neutral Sphingomyelinase inhibitor and is believed to the inhibitor for exosomes. The result by transmission electron microscope showed that GW4869 group had suppressed exosomes in CAFs. Meanwhile, our study also showed that NFs and GW4869 groups showed no significant difference on cell proliferation. Recently, mounting evidence has shown that CAFs enhance the development in several tumor types [21–23], through either re-modifying the TME or regulating the gene expression of the tumor cell itself. Here, we manifested that CAFs secreted exosomes potentiated CRC cell proliferation, invasion, migration and even EMT progression, indicating that CAFs are one of the motivators for CRC cell progression. Our finding of CAFs being responsible for CRC progression agrees with several previous reports [24, 25]. Therefore, CAFs might be a promising therapeutic target for CRC prognosis.

Dysregulation of lncRNAs in cancer cells that involved in cancer growth and progression has been widely reported [26–28]. Besides, lncRNAs in CAFs are increasingly recognized to be important elements for tumor development in multiple solid tumors [29, 30]; however, the role of LINC00659 in CAFs is unclear. The present study showed that LINC00659 is overexpressed in CAFs and CAFs-exo of CRC compared with NFs. Importantly, CAFs transferred exosomes to CRC cells, resulting in an increase of LINC00659 in CRC cells. Further study presented that LINC00659 can be transferred to CRC cells to induce cell proliferation, migration and invasion, indicating that LINC00659 is the mediator participating in CAFs and CRC cell communications. Moreover, TCGA database showed that LINC00659 expression was closely correlated with the prognosis of CRC patients. This observation was in consistent with previous evidence [8]. Previous study reported that LINC00659 aggravates the progression of CRC via multiple mechanisms [8]. As distinguished from other studies of LINC00659 functioning in tumor cells, our study focused on the function of LINC00659 in assisting CAFs to regulate CRC, indicating that targeting LINC00659 or blocking its transport from CAFs to CRC cells could be a possible strategy for CRC treatment. However, there is one ambiguous point that we have not elucidated. Although LINC00659 has been verified to be an important role in CRC by CAFs, the target genes participating in the LINC00659-mediated regulation of cell development remain unknown.

Recently, increasing evidence has shown that lncRNAs played a distinctive role by acting as ceRNAs in tumor progression [31, 32]. In this regards, the current report was then undertaken to explore the underlying ceRNA network underlying LINC00659. Initially, we found that CAFs-exo could suppress miR-342-3p to promote ANXA2 expression. Then, Starbase and dual luciferase reporter assays corroborated that exosomal LINC00659 and ANXA2 were verified to directly target miR-342-3p. Study has shown that miR-342-3p acts as a tumor suppressor [33], and was downexpressed in CRC [34]. In addition, ANXA2 is strongly expressed in pancreatic cancer and facilitates pancreatic cancer progression [35], and literature has also reported the implication of ANXA2 in the prognosis and diagnosis of CRC [36]. Moreover, silence of ANXA2 suppresses TGF-β induced CRC cell invasion, while inhibition of Src/ANXA2/STAT3 could reverse EMT progression [37]. Although the implication of miR-342-3p in CRC cells was reported in a previous study [38], the possible interaction of miR-342-3p and ANXA2 on development of CRC cells have not yet been cleared. Our findings uncovered that exosomal LINC00659 from CAFs down-regulated miR-342-3p and upregulated expression of ANXA2, which contributed to the facilitation of CRC cell development and EMT. For the above observations, we have ultimately deduced that exosomal LINC00659 can act as a ceRNA via sponging miR-342-3p to regulate ANXA2 in CRC cells.

## Conclusion

In summary, we provide evidence that CAF-derived exosomes transport LINC00659 to CRC cells and consequently contribute to CRC cell progression. The mechanism regarding exosomal LINC00659 to promote CRC cell growth may involve miR-342-3p/ANXA2 axis. We envision that blocking the role of exosomal LINC00659 secreted by CAFs might be a potential biomarker and therapeutic target for the prediction and treatment of CRC.

## Supplementary Information

**Additional file 1.** No red fluorescence can be observed in PBS treated LoVo and SW48 cells

## Data Availability

The datasets used or analyzed during the current study are available from the corresponding author on reasonable request.

## Abbreviations

CAFs: Cancer-associated fibroblasts
CAFs-exo: CAFs-derived exosomes
CRC: Colorectal cancer
NFs: Normal fibroblasts
NFs-exo: NFs-derived exosomes
EMT: Epithelial mesenchymal transformation
TME: Tumor microenvironment
lncRNAs: Long noncoding RNAs
ceRNA: Competing endogenous RNA
FBS: Fetal bovine serum
ANOVA: One-way analysis of variance
