# Supplementary figures and images for: Exosomal LncRNA LINC00659 transferred from cancer-associated fibroblasts promotes colorectal cancer cell progression via miR-342-3p/ANXA2 axis
Source: J Transl Med. 2021 Jan 6;19:8. doi: 10.1186/s12967-020-02648-7 (PMC7789760; doi:10.1186/s12967-020-02648-7)

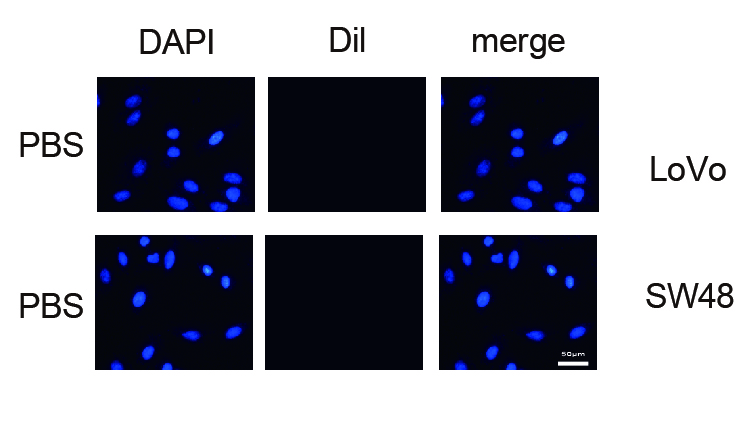

Supplement: Supplementary file 1 — Additional file 1. No red fluorescence can be observed in PBS treated LoVo and SW48 cells [file 12967_2020_2648_MOESM1_ESM.jpg]
